# A model for screen utility to predict the future of printed solar cell metallization

**DOI:** 10.1038/s41598-021-83275-0

**Published:** 2021-02-23

**Authors:** Sebastian Tepner, Linda Ney, Marius Singler, Ralf Preu, Maximilian Pospischil, Florian Clement

**Affiliations:** Fraunhofer Institute for Solar Energy Systems ISE, Heidenhofstraße 2, 79110 Freiburg, Germany

**Keywords:** Energy science and technology, Renewable energy, Solar energy, Photovoltaics, Solar cells, Mechanical engineering

## Abstract

Fine line screen printing for solar cell metallization is one of the most critical steps in the entire production chain of solar cells, facing the challenge of providing a conductive grid with a minimum amount of resource consumption at an ever increasing demand for higher production speeds. The continuous effort of the industrial and scientific community has led to tremendous progress over the last 20 years, demonstrating an average reduction rate for the finger width of approximately 7 µm per year with the latest highlight of achieving widths of 19 µm. However, further reductions will become a major challenge because commonly used metal pastes are not able to penetrate arbitrary small screen opening structures. Therefore, this study introduces the novel dimensionless parameter screen utility index SUI which quantifies the expected printability of any 2-dimensional screen architecture in reference to a given paste. Further, we present a full theoretical derivation of the SUI, a correlation to experimental results and an in-depth simulation over a broad range of screen manufacturing parameters. The analysis of the SUI predicts the point when commonly used wire materials will fail to provide sufficient meshes for future solar cell metallization tasks. Therefore, novel wire materials (e.g. the use of carbon nanotubes) with very high ultimate tensile strengths are discussed and suggested in order to fulfill the SUI requirements for printing contact fingers with widths below 10 µm. We further analyze economic aspects of design choices for screen angles by presenting an analytical solution for the calculation of mesh cutting losses in industrial screen production. Finally, we combine all aspects by presenting a generalized approach for designing a 2-dimensional screen architecture which fulfills the task of printing at a desired finger width.

## Introduction

**Figure 1:**
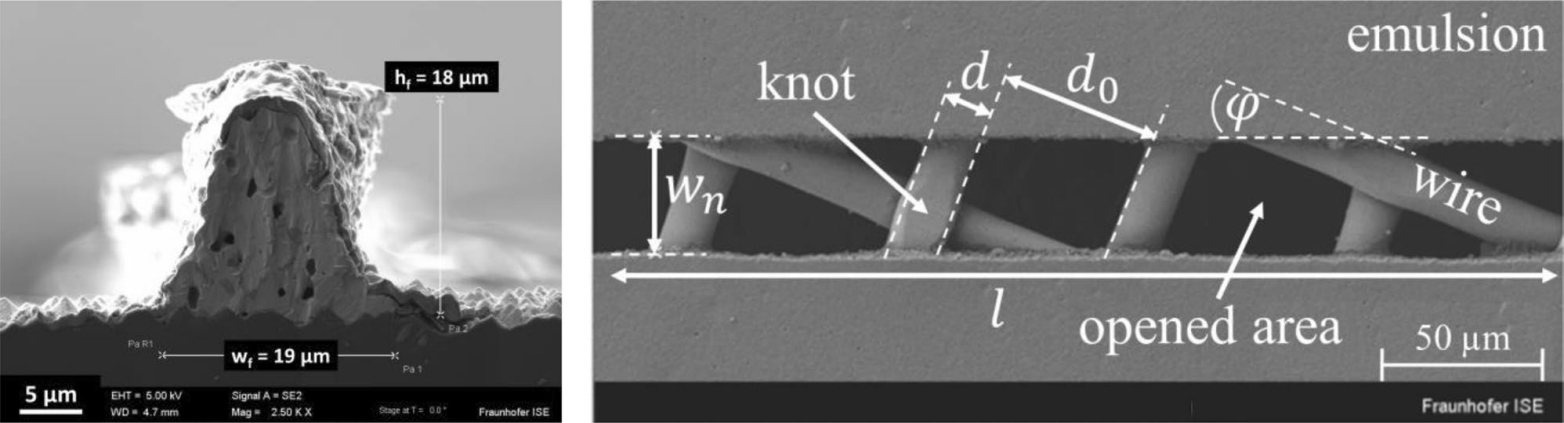
On the left, a fine line Ag-electrode (finger) for Si-solar cell metallization is shown^16^. On the right, we present a SEM image of a screen opening channel, defined by the nominal screen opening width ${\text{ w}}_{{\text{n}}}$, the channel length ${\text{ l}}$, the wire diameter ${\text{ d}}$, the mesh opening ${\text{ d}}_{0}$ and the angle between emulsion edge and mesh wires $\varphi$. The crossover area of two wires is defined as a so-called knot and the black areas represent the individual opened areas of the channel^13^.

Today’s metallization of Silicon solar cells is still dominated by flatbed screen printing^1^ mainly because of its reliable and cost-effective production capabilities. Within the last two decades, the scientific community has made tremendous progress in reducing the finger width from approx. 100 µm in 2006^2^ to only 26 µm on cell level published by Tepner et al. in 2019^3^. In the same year, we were able to reduce the printed finger width down to approx. 20 µm at a record aspect ratio of approx. 0.95 on a test layout as presented in Fig. 1 on the left^4,5^. This trend of approx. 7 µm per year was mainly driven by independent paste and screen optimization. Thibert et al. presented a comprehensive study on how the rheological behavior of Ag-paste is influencing the screen printing performance. Pospischil et al. further related common rheological parameters of metal pastes to the printed finger geometry, showing the dominant impact of the yield stress on the aspect ratio^6–8^. Furthermore, Xu et al. showed for specially designed paste formulations, how wall slip at the emulsion surface can significantly improve the paste transfer. Tepner et al. expanded on this idea by generalizing the method for the analysis of slip phenomena in screen printing. Further, they demonstrated an improved wall slip behavior of commercially available metal pastes^3,9^. Besides the influence of the paste rheology, the screen architecture itself plays a crucial role when an optimized paste-screen interaction is desired^10^. Figure 1 on the right, illustrates how the final 2-dimensional architecture of a screen is the result of combining a mesh, defined by the mesh count MC and the wire diameter d, and the applied emulsion, defined by the nominal screen opening width w_n_ and the screen angle φ. Previous works have correlated the mesh parameters and the screen opening width with the quality of finger geometry^4,11,12^. Ney et al. introduced a screen simulation approach that is able to predict the exact size, shape and location of all individual opened areas within a screen opening based on the presented four parameters^13,14^. Tepner et al. has later correlated this simulation approach to experimental data^4^. Furthermore, they have expanded this simulation approach to determine how the number of wire crossings within the screen opening depends on the presented four screen parameters. Based on these results, they were able to derive specific screen angles to create knotless configurations (zero wire crossing), suggesting an increased screen lifetime and improved printability at the same time^15^. On the other hand, White et al. and Taroni et al. presented a comprehensive mathematical model for the mechanics of the screen printing process which could be used for further optimization attempts. However, to this date, the application of these models is quite challenging, missing clear and easy to use design goals for screen production in a volatile PV market.

For this reason, we will present a generalized theory for the design of a 2-dimensional screen architecture by deriving a dimensionless parameter which describes the impact of the screen utility during the printing process. In the following section, we will first summarize the theoretical background for screen design and then expand on this background by deriving the screen utility index SUI. Furthermore, we will show experimental verification of the presented simulation approach by comparing the simulated area of individual openings to microscope images of different screen architectures. Finally, we present a comprehensive simulation of the dependency of the SUI on all 2-dimensional screen parameters. This data will allow the industry to improve their decision-making process for novel screen configurations without requiring complex mathematical modeling of the process mechanics. The goal of the presented approach is to further improve the metallization of Si-solar cells in mass production in terms of increased cell efficiency and reduced production cost. Especially, the reduction of silver consumption per cell by an improved fine-line screen printing process is crucial when facing the predicted silver production crisis, when the demand of the PV industry for silver will exceed worldwide silver production by the year 2030^17^.

## Theoretical background

This section summarizes briefly the state of the art parameters to describe the overall quality of a screen in terms of its printability and life time in a production environment. The four introduced parameters (mesh count MC, wire diameter d, screen opening width w_n_ and the screen angle φ) define the resulting 2-dimensional screen opening channel. Further they can be used to derive more specific measures for the screen quality. The normalized open area OA_%_ is the most established parameter to characterize the geometrical architecture. It is defined by the ratio of opened area to the overall area of one mesh unit and can be calculated by Eq. (1)^18^.

1 $$ {\rm{OA}}_{\% } = \frac{{{\rm{d}}_{0}^{2} }}{{({\rm{d}} + {\rm{d}}_{0} )^{2} }}. $$

It shall be noted that this parameter describes the average value across an infinitely long screen opening channel. We are not aware of an analytical solution which describes the local deviation of OA_%_ across the length of the screen opening channel and its dependency on the screen angle. As discussed in the introduction section, we have previously published an approach which is able to simulate this local deviation across the screen opening σ_OA_^13^.

The wire-to-wire distance ${\text{d}}_{0}$ is calculated by Eq. (2) with the mesh count MC, describing the number of wires per unit length, and the wire diameter d^18^.

2 $$ {\text{d}}_{0} = 1{\text{ / MC}} - {\text{d}}{.} $$

One important parameter to ensure sufficient printability over the maximal possible screen life cycle is the screen tension γ_screen_. Depending on the mesh count MC and the wire diameter d, a maximal possible screen tension γ_screen_max_ can be given by Eq. (3)^18^.

3 $$ \gamma_{{{\text{screen\_max}}}} = \sigma_{{{\text{uts\_wire\_mat}}}} \cdot {\text{MC}} \cdot \pi \frac{{{\text{d}}^{2} }}{4}. $$

The ultimate tensile strength of a single mesh wire σ_uts_wire_max_ is a material parameter, describing the minimal stress which is necessary to break the material while being stretched. The state of the art material for mesh wires in the PV industry is stainless steel with an ultimate tensile strength σ_uts_wire_max _≈ 800 N/mm^2^^19^. Recently, the use of tungsten alloy wires emerged in order to produce mesh with wire diameters below 15 µm^4,5,14^. Horwarth et al. analyzed the impact of a reduced screen tension γ_screen_ on the screen lifetime, making it one of the most important parameters for the industry^20^. Further publications deal with 3-dimensional parameters, e.g. emulsion height EOM and the calendaring of the mesh, showing an additional influence on the printing performance^6,11,12,21–23^. However, the presented generalization of the screen performance will only rely on a 2-dimensional analysis, allowing the reader to compare any screen configuration with the same set of 3-dimensional parameters.

## Methods

### Simulation approach

In this study, a full simulation of all individual opened areas across a screen opening channel w_n_ has been carried out. The mathematical background of this simulation approach is well described in literature^4,5,13,15^. The area heavily depends on the screen manufacturing parameters e.g. screen opening width w_n_, screen angle φ, mesh count MC and wire diameter d, leading to a full parameter sweep presented in Table 1. In total, all possible combinations of 68,600 different mesh configurations, 450,000 different screen angles φ and 3400 different screen opening width w_n_ are simulated. For all screen opening channels, a length of l = 156 mm is assumed. Furthermore, the position on the screen orthogonal to the screen opening is averaged over 100 screen openings.

**Table 1:** Variation of parameters for the simulation of the area of individual opened areas across a screen opening. The length of all simulated channels was set to the industry standard of 156 mm (2020)^3^. The position on the screen orthogonal to the screen opening is averaged over 100 screen openings.

Parameter	Start	Increment	End
Mesh count MC (inch)	100	10	1500
Wire diameter d (µm)	1.0	0.1	50.0
Screen Angle φ (°)	0.0000	0.0001	45.0000
Screen opening width w_n_ (µm)	5.00	0.01	40.00

### Experimental verification of the simulation approach

The area of individual openings of two commercial available screens with a screen angle of φ = 22.5° are evaluated by microscopy. The first screen has a mesh count of MC = 360 1/inch, a wire diameter of d = 16 µm and a screen opening width of w_n_ = 40 µm. The second screen has a mesh count of MC = 380 1/inch, a wire diameter of d = 14 µm and a screen opening width of w_n_ = 30 µm. For both screens, a 1 mm segment in the center between busbars of the 25th, 50th and 75th screen opening of the layout is investigated by measuring the area of all individual openings and determining its shape. Afterwards, the exact screen opening segments are simulated to obtain the simulated area of individual openings which are then compared to the experimental data. Furthermore, we presented an additional verification of the simulation approach by comparing the screen angle dependency of individual opened areas, demonstrating an completely accurate simulation of their size^13^. Later, we expanded the simulation approach by simulating the amount of different classes of wire crossings across the screen opening channel and comparing them to microscope images, proving the precise prediction capability of the presented screen simulation approach^15^.

## Results and discussion

### Introduction of the screen utility index

**Figure 2:**
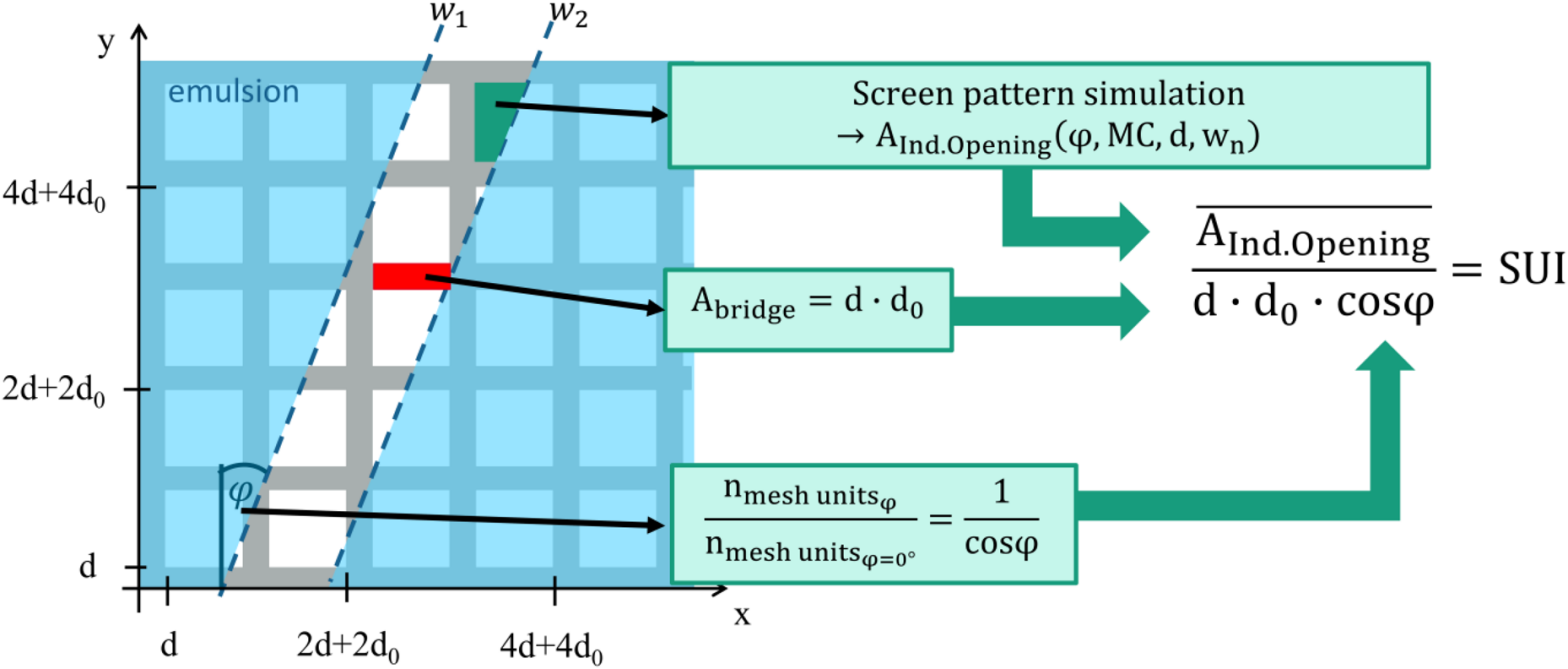
Definition of the screen utility index SUI, describing the relationship between the area of individual openings and the area of individual mesh bridges weighted by the amount of mesh units per screen opening channel.

In order to generalize the screen opening pattern, a relationship between the screen manufacturing parameters (e.g. screen opening width w_n_, mesh count MC, wire diameter d and the screen angle φ) and the resulting opening pattern needs to be derived. Figure 2 presents a way to deconstruct the screen opening channel, resulting in a single dimensionless parameter which describes the general utility of a screen. The screen utility index SUI is constructed in three parts:

1. The average area of an individual opening defines how much fluid is able to transfer through the screen opening channel w_n_ at given pressure. An analytical solution for the dependency of this area A_Ind.Opening_ on all described screen manufacturing parameters is unknown and therefore, requires the presented simulation approach. The average size of all openings is directly linked to the printability.
2. In order to quantify the impact of the underlying mesh on the screen performance, the area of a single mesh bridge A_bridge_ is defined by Eq. (4). Using a fine mesh (high mesh count MC or small wire to wire distance d_0_) will increase the screen lifetime due to an increased wire intersection coverage^15^. Furthermore, the screen tension is increased, improving the screen snap-off mechanics to minimize spreading effects^15,18^. On the other hand, the wire diameter d should be minimized because the paste transfer is strongly limited by a blocking cylindrical object. Whitney et al. analyzed the force–velocity relationship of a rigid cylinder moving through a highly non Newtonian fluid, showing that commonly used metal pastes with a flow index n ≪ 1 require forces more than one order of magnitude higher than for Newtonian flows with equal velocities and geometric conditions^24,25^. This scenario applies directly to the screen printing process during screen snap-off and indirectly to the flooding phase^3^. Combining both statements for the wire-to- wire distance d_0_ and the wire diameter d, will lead to the conclusion to minimize the area of a mesh bridge A_bridge_.
  4 $$ {\text{A}}_{{{\text{bridge}}}} = {\text{d}}_{0} \cdot {\text{d}}{.} $$
3. The dependency of the amount of contributing mesh units within a screen opening channel on the screen angle φ is presented in Eq. (5). Due to this relationship, the ratio between the average area of individual openings A_Ind.Opening_ and the area of single mesh bridges A_bridge_ must be multiplied by the corresponding factor 1/cos(φ) in order to account for the decreased angle dependent number of mesh units per screen opening channel at nonzero angles. Each mesh bridge contributes to the expected stability of the emulsion edge during printing because it acts as a micro foundation.
  5 $$ \frac{{{\text{n}}_{{{\text{mesh \;units}} \varphi }} }}{{{\text{n}}_{{{\text{mesh\; units}} \varphi = 0^\circ }} }} = \frac{1}{\cos \varphi }. $$

Finally, the definition of the dimensionless screen utility index SUI can be given in Eq. (6) by combining the presented three statements. Any screen configuration, defined by its 2-dimensional geometric parameters has one specific screen utility index SUI. However, there is an infinite amount of theoretical screen configurations which result in the exact same value of the SUI.

6 $$ {\text{SUI}} = \frac{{\overline{{{\text{A}}_{{{\text{Ind.Opening}}}} }} }}{{\cos \varphi \cdot {\text{d}} \cdot {\text{d}}_{0} }}. $$

Following this statement gives rise to a classical optimization problem. What value for the SUI is good enough to ensure printability in respect to a fixed reference fluid or paste? In order to answer this question, we must analyze the special case where SUI = 1 applies. In that case, the average size of an individual opening is equal to the area of a blocking wire bridge, weighted by the amount of mesh units across a screen opening channel. This relationship puts the impact of the mesh into context to the resulting screen opening channel, finding a balance between a fine mesh, optimized for high screen tension as well as the screen life time, and the task of providing a sufficient paste transfer at the desired screen opening width w_n_. In the regime where SUI < 1 applies, the chosen underlying mesh is too coarse for the desired w_n_, resulting in a significant limitation of the fluid transfer. The relationship between the different screen parameters and the resulting SUI is nonlinear because the nominator is depending on all screen parameters itself. For example, increasing the mesh count MC (decreasing the wire to wire distance d_0_) would increase the SUI. However, the average area of individual openings will decrease at the same time, resulting in a highly nonlinear reduction of the SUI. On the other hand, in the regime where SUI > 1 applies, the underlying mesh is fine enough to create a sufficient screen opening pattern and therefore not limiting the paste transfer more than the angled screen opening channel width w_n_ would have done anyway. It must be noted that the special case of SUI = 1 mainly applies for a homogeneous fluid which is either particle free or contains a particle size distribution where the majority of particles are small compared to the individual opening size. Therefore, we suggest a threshold for the ratio between the size of the majority of particles (e.g. particles with a diameter smaller than d_99%_, assuming a normal distribution) and the individual average opened area in Eq. (7).

7 $$ \frac{1}{4}\pi {\text{d}}_{99}^{2} \ll \overline{{{\text{A}}_{\text{Ind.Opening}} }} . $$

If the d_99%_ value of a highly filled suspension (e.g. metal pastes for solar cell metallization) becomes too big, certain small openings included in the nominator of Eq. (6) are not able to contribute anything to the overall paste transfer due to immediate clogging by individual particles or agglomerates. As soon as this effect cannot be neglected anymore, the threshold of the minimal required SUI value for a sufficient printability (SUI_min_) becomes a function of the clogging probability itself. At this point, we are going to suggest an empirical value for SUI_min_ for commonly used high temperature Ag-paste for PERC front-side metallization in the following experimental section. In order to further model the correlation between the SUI_min_ and the clogging probability of individual opened areas within a screen opening channel, an experimental method to measure the clogging event during the screen printing process needs to be developed. At this point, the mechanics of the screen printing process do prevent an easily accessible method for direct measurements.

### Predictability of the simulation approach

**Figure 3:**
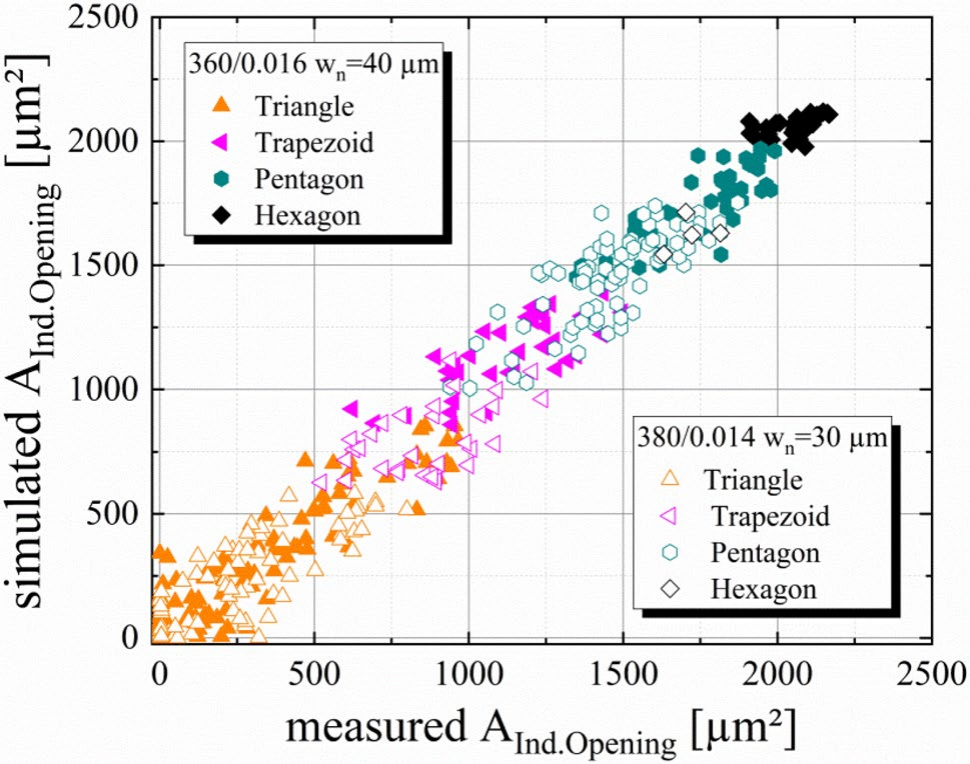
Experimental verification of the simulated area of individual openings. For this verification, 27 microscope images per screen of the screen opening channels were taken and analyzed regarding the area of individual openings. Two screens with different mesh types are chosen. The first mesh has a mesh count MC = 380 1/inch and a wire diameter d = 14 µm (open symbols) with w_n_ = 30 µm and the second mesh has a mesh count MC = 360 1/inch and a wire diameter d = 16 µm with w_n_ = 40 µm (closed symbols). The screen angle of both screens is φ = 22.5°. The deviation of measurements is caused by manufacturing tolerances of ${\text{w}}_{{\text{n}}}$, MC, d and $\varphi$, the mesh calendaring and an insufficient resolution of microscope images.

In Fig. 3, we present the experimental verification of the simulation approach by comparing simulated values for the area of individual openings to measurements of the corresponding area by microscope images. As described in section “Experimental verification of the simulation approach”, two different commercial available screens with a screen angle φ = 22.5° are used. The first screen has a mesh count MC = 360 1/inch with a wire diameter d = 16 µm (w_n_ = 40 µm) and the second screen is made out of a mesh, using a MC = 380 1/inch with a wire diameter d = 14 µm (w_n_ = 30 µm). The presented deviation between measured and simulated sizes for the exact same individual opening is not caused by the simulation approach itself, rather than resolution limitations of microscopy. Furthermore, manufacturing tolerances of w_n_, d, φ and further deviations due to the mesh calendaring are causes for the deviation. However, the overall predictability of the presented simulation approach for the area of individual openings is verified and shall be used to obtain values for the presented dimensionless parameter screen utility index SUI because the area of individual openings is the only parameter within Eq. (6) which must be simulated by this model.

### Correlation of the SUI with screen printing experiments

**Figure 4:**
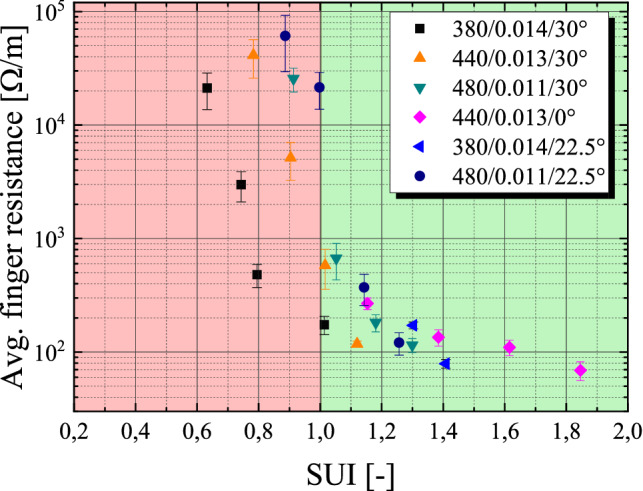
Correlation of the average finger resistance on the SUI value. The predicted change of printability at a SUI = 1 is supported by experimental data. For values where SU I < 1 applies, the underlying mesh will have an over proportional negative influence on the printing performance. On the other hand, for values where SUI > 1 applies, the mesh will not limit the paste transfer more than natural limitation of the screen opening channel w_n_. Different screens are plotted for 24 µm, 21 µm, 18 µm and 15 µm screen openings. The data is taken from our previous publication^4^. Values for the 380/0.014/22.5° screen are taken from^3^.

In Fig. 4, we present experimental data from our previous publication^4^, demonstrating the impact of the SUI on screen printed metallization by increased lateral finger resistance R_L_. For this experiment, the paste sample, the emulsion height EOM, the rate of calendaring of the mesh and all printing parameters has been kept constant. In “Results and discussion”, we have discussed the theoretical meaning of SUI = 1, highlighting the change when the mesh starts to contribute significantly to the limitation of further paste transfer. The presented data supports this critical point where SUI = 1 applies and further shows that even approaching SUI = 1 will have consequences in terms of significant increase of the lateral finger resistance R_L_ and thus reduced cell efficiency and non-optimal silver consumption. In order to understand this, we elaborate on the underlying optimization problem of solar cell metallization. The shading of the active cell area by the metallization grid is determined by the cell layout (e.g. number of busbars), the interconnection concept, the finger geometry (mainly the width) and the number of fingers. An increase in shading losses directly results in a significant reduction of the short circuit current density J_sc_ and subsequently solar cell efficiency. As these shading losses of the grid should be minimized, one must also consider the series resistance contribution of the grid^26^. Here, the lateral finger resistance as well as the contact resistance at the metal–semiconductor contact plays a crucial role. The latter is mainly determined by paste formulation, configuration of the firing process as well as actual properties of the solar cell precursor. However, on the other hand, the lateral finger resistance for a given paste is predominantly determined by the geometry of the printed structure and therefore strongly correlates with printing results. The finger resistance increases whenever the cross-sectional area of a contact finger is locally reduced across its length due to insufficient printing. In our previous publication we calculated the maximal tolerable lateral finger resistance for different interconnection concepts when a maximal finger series resistance contribution of r_s_ = 0.1 Ω cm^2^ is assumed^27^ showing that there is hard limit for the maximal tolerable lateral finger resistance per given cell layout and interconnection concept. On top of that, the overall goal for the metallization process remains always to minimize silver consumption while meeting the described performance requirements. When we now come back to the screen utility index SUI, we must consider the correlation between the SUI and experimental data for the lateral finger resistance for each paste separately. As discussed in section “Introduction of the Screen Utility Index”, if one uses a highly filled suspension for which Eq. (7) does not remain true, the margin for minimal SUI shifts towards higher levels because the effective average area of individual openings is reduced due to clogging by single particles or agglomerates. Based on the presented data in Fig. 4, we suggest for high temperature Ag-pastes (HT-Ag), used for PERC front-side metallization, a minimal threshold of SUI_min_ (HT-Ag) = 1.25. Furthermore, for low temperature Ag-paste (e.g. metallization of HJT solar cells) we predict a suitable margin of SUI_min_ (LT-Ag) > 1.6 and for Al-paste for rear side metallization of bifacial PERC, we predict a necessary margin of SUI_min_ (HT-Al) > 1.9. However, to this date, there is no specific evidence for the last two predictions. Furthermore, we would like to point out that small deviations on screen configurations due to manufacturing tolerances might cause a deviation of the SUI, negatively (or positively) influencing the expected printing result further. In future studies, these deviations should be experimentally investigated in order to directly link manufacturing tolerances to a potential reduction in printability.

**Figure 5:**
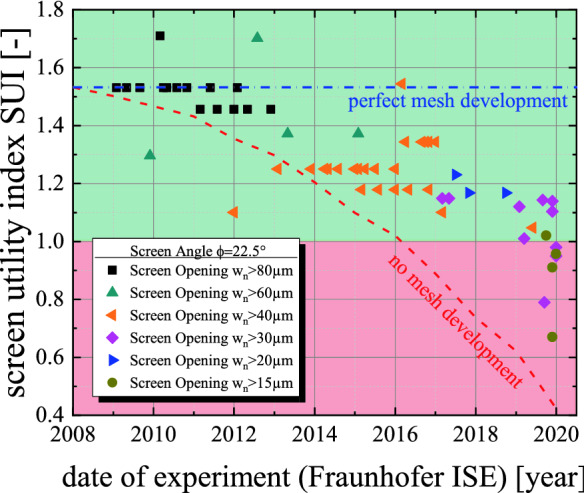
History of screen printing experiments at Fraunhofer ISE using screens with the shown screen utility indices SUI. The progression towards smaller SUI values shows the natural evolution of the fine line screen printing process for metallization of Si-solar cells. The nominal screen opening width was continuously reduced over the years. The development of finer mesh patterns was not able to keep up with this trend, resulting in an average SUI reduction of approx. 0.05 points per year. In 2019, Fraunhofer ISE has challenged the screen printing process with an intense reduction of screen opening structures down to 15 µm.

In Fig. 5, we present accumulated data from successful screen printing experiments at Fraunhofer ISE (Freiburg, Germany) for Si-solar cell metallization over the last ten years, demonstrating the evolution of the SUI in a research environment. A variety of different mesh counts MC and wire diameters d has been used to ensure printing through an ever decreasing screen opening width w_n_. However, without realizing it at the time, the SUI has been reduced over the years, indicating that mesh manufactures where not able to keep up their development of finer meshes with the reduction rate of the screen opening width *d*w_n_/*d*t. Nevertheless, the absolute value for the SUI over the years was still suitable for mass production because not even the SUI = 1.25 limit was passed. This offers a potential explanation why the evolution of published results for printed finger width over the last 15 years was achieved at an outstanding reduction rate of more than 7 µm per year^28^. Further paste development was enough to drive this evolution as SUI values during that time span were far beyond SUI > 1.25, revealing that the screen was never the limiting factor when it comes to printability. In Fig. 7, we present the gap between the constant blue line and actual evolution of the SUI, giving a qualitative measure for this contribution of the paste development. Those improvements on the paste printability were able to compensate the (at the time) hidden reduction of the SUI. On the other hand the red line shows the theoretical evolution of the SUI when no mesh improvements since 2010 would have been achieved at all. The gap between this trend and the actual evolution gives a qualitative measure for the mesh development. Especially in recent years, the paste development has an increasing impact on the further reduction of the achieved finger width.

Furthermore, we would like to highlight the fact that the overwhelming industry standard for the screen angle φ = 22.5° was dominating even the research activities in a way that almost no data for different screen angles φ are available^29–33^. Only in recent years so 0° knotless screens and 30° angled screens have been investigated^4,34^. Figure 5 further reveals that in recent years we have challenged the screen printing process to the point where usual screen architectures fail completely. In 2019, significant reductions of the screen opening width w_n_ from initial 27 µm towards a novel test pattern with screen openings ranging from 24 µm to only 15 µm have cut the resulting SUI almost in half. This result highlights how the mesh development is a critical step of overall screen development. Therefore, the rate in which screen manufactures decrease the screen opening width w_n_ should not be done as quickly as possible as it requires a strong communication with mesh manufactures beforehand.

### Simulation of the SUI

#### Optimization of the mesh count MC and wire diameter d

**Figure 6:**
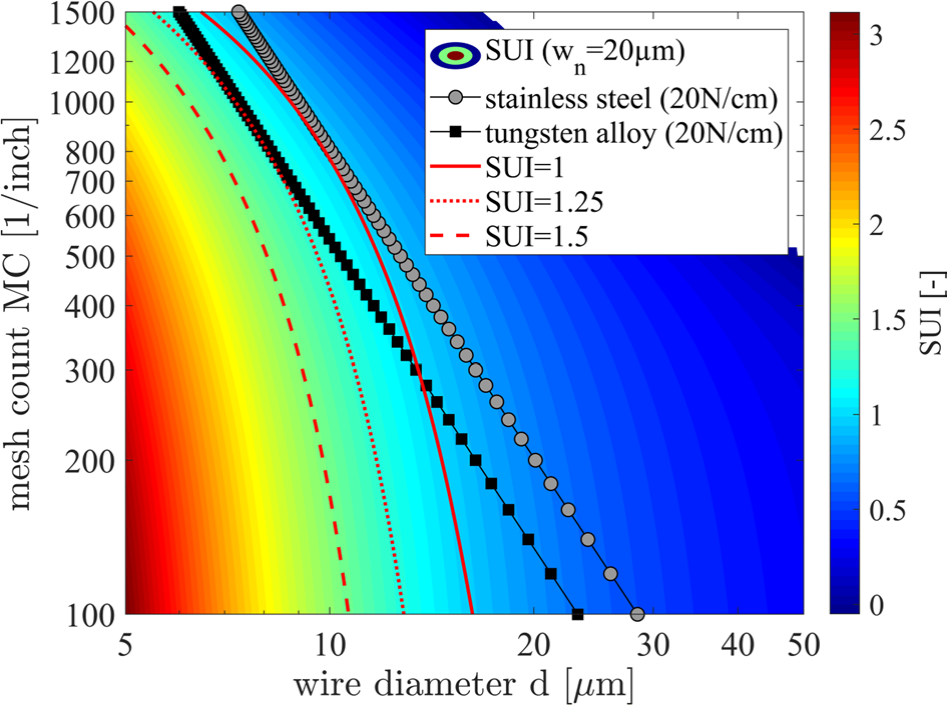
The SUI is simulated for 68,600 different mesh configurations with a screen opening width of w_n_ = 20 µm at a screen angle of φ = 22.5°. In red, constant SUI lines are presented, highlighting the minimal threshold at SUI = 1. Further, the SUI = 1.25 line is shown, indicating the minimal barrier for a sufficient printability of commonly used Ag-pastes. The black and grey lines indicate a constant screen tension for tungsten alloy and stainless steel wires at γ_screen_ = 20 N/cm. The intersection point of the SUI = 1.25 line with the screen tension function defines the optimal mesh choice.

Figure 6 shows the SUI dependency on both mesh parameters for a screen opening width of w_n_ = 20 µm with a screen angle φ = 22.5°. As discussed in section “Introduction of the Screen Utility Index”, the nominator of the SUI is depending on the wire to wire distance d_0_ and the wire diameter d itself, resulting in the presented nonlinear relationship between the SUI and the mesh parameters. This result gives rise to a classical optimization problem because a mesh with a very low mesh count MC and a small wire diameter d will maximize the SUI, but at the same time minimizes the screen stability due to Eq. (3). In order to highlight this circumstance, we have added red curves for constant SUI values as well as curves for the maximal possible screen tension γ_screen_max_ = 20 N/cm for stainless steel and tungsten alloy wires. The intersection of a constant SUI line (e.g. SUI_min_ = 1.25) with the curve for the constant screen tension gives the minimal requirement for the mesh in terms of minimal mesh count MC and maximal wire diameter d at which the SUI > 1.25 threshold is fulfilled. If an intersection point between the constant SUI line and the maximal possible screen tension curve for a given wire material does not exist, the desired configuration is physically impossible. In such a case, a new wire material with an increased ultimate tensile strength σ_uts_wire_mat_ needs to be developed. This approach reveals the threshold at which a given wire material with an ultimate tensile strength σ_uts_wire_mat_ is able to fulfill the requirements for a screen with a certain screen tension γ_screen_ (as long as γ_screen_ < γ_screen_max_ remains true) and the desired value for the SUI which fulfills SUI > SUI_min_.

**Figure 7:**
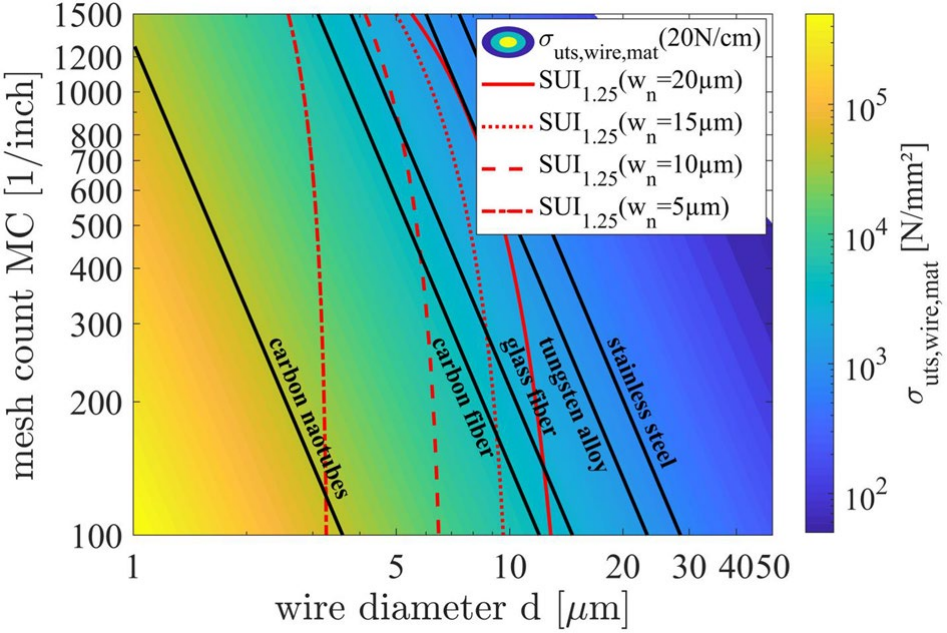
The simulation of the ultimate tensile strength σ_uts_wire_ of individual wires. The mesh parameters are varied between 100–1500 1/inch for the mesh count MC and 1–50 µm for the wire diameter d. A fixed screen tension of 20 N/cm was used. Furthermore, constant lines for certain materials are shown and discussed (e.g. stainless steel, tungsten, glass fibers, carbon fibers, carbon nanotubes). Finally, constant SUI lines are given for different desired screen opening channels w_n._

In Fig. 7, we present a suggestion for future wire materials by plotting Eq. (3) for a broad range of mesh counts MC and wire diameters d with a screen tension γ_screen_ = 20 N/cm. Furthermore, we are adding constant SUI lines which highlight the need for further developments of novel wire materials because commercial available wire materials like stainless steel and tungsten alloys already show very limited capabilities for further reduction of the screen opening width w_n_. For example, there exist different types of fiber glass with high ultimate tensile strengths which could be a suitable option for woven mesh wires^35^. Usually, the glass of such fibers is amorphous, providing a homogenous structure along and across the fiber, however the production of these fibers at diameters below 10 µm is challenging, because even small scratches on the surface will dramatically influence the mechanical properties^36^. On the other hand, there exist fibers made out of carbon. They are widely used in the industry to produce strong and ultra-light components for a broad range of applications. Kumar et al. tested single carbon fibers with diameters down to 7 µm, reporting ultimate tensile strengths of up to 3200 MPa^37^. Arshad et al. produced carbon fibers with an electrospinning approach, with diameters below 0.5 µm and an ultimate tensile strength in the range of 4500 MPa^38^. Finally, if we further examine the thought experiment of using the finest possible “wire” with the maximal obtainable ultimate tensile strength, we will eventually arrive at Iijima and Ichihashi, who published the discovery of carbon nanotubes in 1993^39^. Takakura et al. measured for the first time the ultimate tensile strengths of an individual structure-defined, single-walled carbon nanotube with values for the ultimate tensile strength ranging from 20 GPa to over 50 GPa^40^. Furthermore, Zhang et al. was able to produce over 50 cm long carbon nanotubes by a floating chemical vapor deposition process in 2016^41^, making an industrial application for mesh production potentially a matter of years rather than multiple decades. The ultimate tensile strength of one these potential wire or fiber materials need to be higher than the minimum requirement for the desired SUI. For example, if a screen with a nominal screen opening channel of only w_n_ = 5 µm is manufactured, the underlying mesh cannot be made out of conventional wires. New technologies for the mass production of very thin and strong wires or fibers (e.g. carbon nanotubes) have to be developed in order to prevent an upcoming dead end of ultra-fine line metallization of Si-solar cells.

#### Optimization of the screen angle φ and screen opening width w_n_

**Figure 8:**
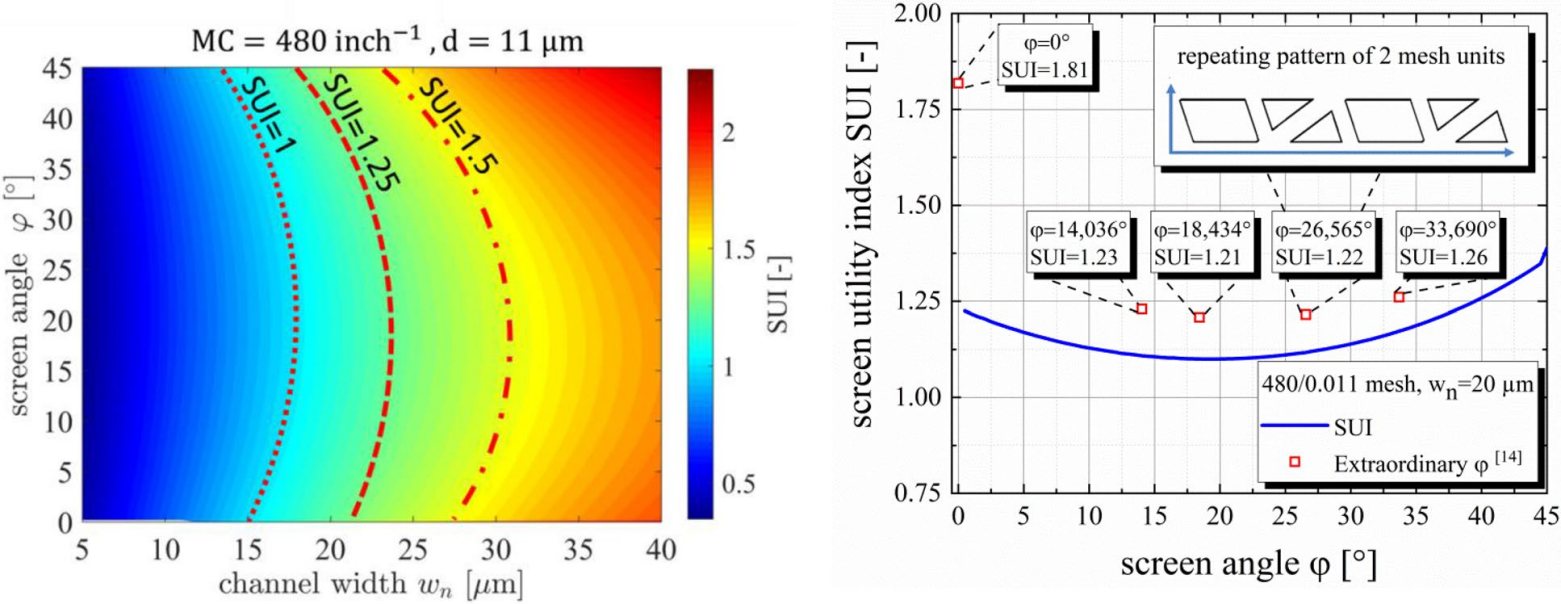
On the left, the screen utility index is simulated for all possible screen angles—screen opening width w_n_ combinations between 0° < φ < 45° and 5 µm < w_n_ < 40 µm. The underlying mesh is kept constant with a MC = 480 1/inch and d = 11 µm. The constant SUI lines for SUI = 1, 1.25, 1.5 are highlighted in red, indicating the nonlinear dependency on the screen angle φ. For low screen angles, the nonlinear characteristic vanishes whereas for high angles close to 45° the dependency becomes the dominant influencing factor. On the right, the screen utility index for the 480/0.011 mesh is highlighted with the inclusion of extraordinary screen angles φ. In our previous publication we derived those angles by utilizing the Farey sequence, revealing a highly repetitive opening pattern^15^. If those angles are compared to the average angle dependency of the SUI, a significant increase can be observed.

In Fig. 8 on the right, a full simulation of the SUI for screen angles between 0°–45° and screen opening channel width w_n_ between 5 and 40 µm are shown, revealing a nonlinear relationship between the SUI and the screen angle φ. Red lines highlight constant SUI curves, including the SUI = 1.25 margin discussed earlier. The common industry standard of φ = 22.5° results in one of the worst configurations if a reduction of w_n_ is desired. The screen manufacturer should switch to reducing or increasing the screen angle to avoid the regime where the SUI shows the strongest reduction with further reducing w_n_. However, increasing the screen angle will also increase the total area of mesh per screen required due to increased cutting losses during production. These cutting losses contribute significantly to the overall production costs and should be minimized. The industry produces meshes on weaving machines, creating a “mesh carpet” on a roll with a width of usually 1 m. Afterwards, single sheets of mesh are cut out of this mesh roll.

**Figure 9:**
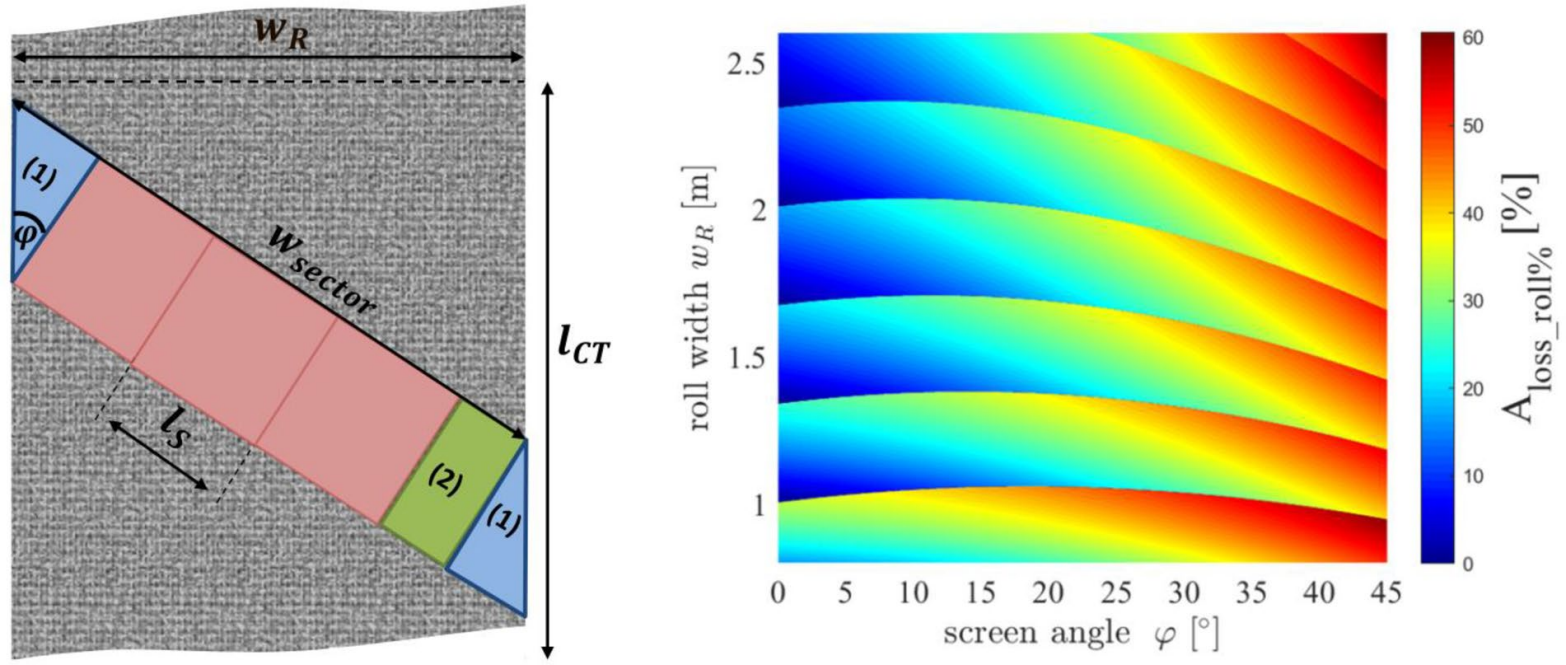
On the left, a schematic illustration for cutting losses of mesh during screen production is presented. Depending on the screen angle φ, the number of sheets with a side length l_s_ which fit into the parallelogram shaped sector is shown. The cutting losses are defined by two triangles with the same size and a remaining rectangle (defined by the residual of sheets fitting into the sector). On the right, a calculation of Eq. (9) for the relative cutting losses of an entire mesh roll is shown for different roll width w_r_ and screen angles φ. The length of the clamping table was set to l_ct_ = 5 m.

In Fig. 9, we present a model to quantify these cutting losses by calculating how many individual square sheets of mesh with side length l_s_ can be cut out of the mesh roll with the screen angle φ. In order to do that, the mesh needs to be clamped on a clamping table with a length of l_ct_. In Eq. (8), we present, to our knowledge for the first time, an analytical solution for the absolute cutting loss per angled sector (see Fig. 9). Further, in Eq. (9) we are presenting a solution for the relative loss of the entire mesh roll based on the roll dimensions, the sheet side length l_s_, the length of the clamping table l_ct_ and the screen angle φ.

8 $$ A_{loss\_sector} = \tan (\varphi ) \cdot l_{s}^{2} + \left( {mod_{{l_{s} }} \left( {\frac{{w_{r} - \sin (\varphi ) \cdot l_{s} }}{{\cos (\varphi ) \cdot l_{s} }}} \right) \cdot l_{s} } \right) $$

9 $$ A_{loss\_roll\% } = \frac{{A_{loss\_sector} }}{{w_{r} }} \cdot \left( {\frac{\cos (\varphi )}{{w_{r} }} - \frac{\sin (\varphi )}{{l_{ct} }}} \right) + \tan (\varphi ) \cdot \frac{{w_{r} }}{{l_{ct} }}. $$

On the right of Fig. 8, a full calculation of Eq. (9) is shown for different roll width w_r_ between 0.8–2.6 m and all screen angles between 0°–45°. At commonly used roll widths of w_r_ = 1 m, the angle dependency of the cutting losses is close to its maximum, suggesting to reduce the screen angle φ as much as possible when economic design goals are considered.

However, as discussed in the following section this creates other serious disadvantages and therefore causes a significant optimization problem for the screen angle φ. We suggest that the industry develops and builds bigger weaving machines in order to increase the scalability of the mesh production. In section “Conclusion”, we will come back to this optimization problem by presenting a comprehensive approach to design any 2-dimensional screen architecture. As mentioned, reducing the screen angle will increase the SUI and therefore the printability, however it will also significantly increase local deviations of the opening area across the screen angle as discussed by Ney et al.^13^. This phenomenon might increase the probability of local finger interruptions for screen angles, especially below φ < 10°. This circumstance shows the complexity of choosing the right screen angle for given screen opening width w_n_. Tepner et al. analyzed specific screen angles which show high repeating pattern of opening structures, showing that for e.g. φ = 26.565° a knotless screen pattern with a repeating pattern every 2 mesh units will occur. This type of knotless screen completely prevents the negative influence of a strong deviation of the opening rate OA_%_ across the screen opening channel. In Fig. 8 on the right, we have specifically simulated the SUI for those screen angles which show outstanding repeating pattern, using the same screen opening channel w_n_ = 20 µm and a mesh with a mesh count MC = 480 1/inch and a wire diameter d = 11 µm. It becomes clear, that a conventional sweep of the screen angle φ even for an increment ∆φ = 10^–4^° is not fine enough to explore the full complexity of the angle depending screen opening pattern. This phenomenon has been discussed in our previous publication in more detail^15^.

### Optimization approach for future screen design

**Figure 10:**
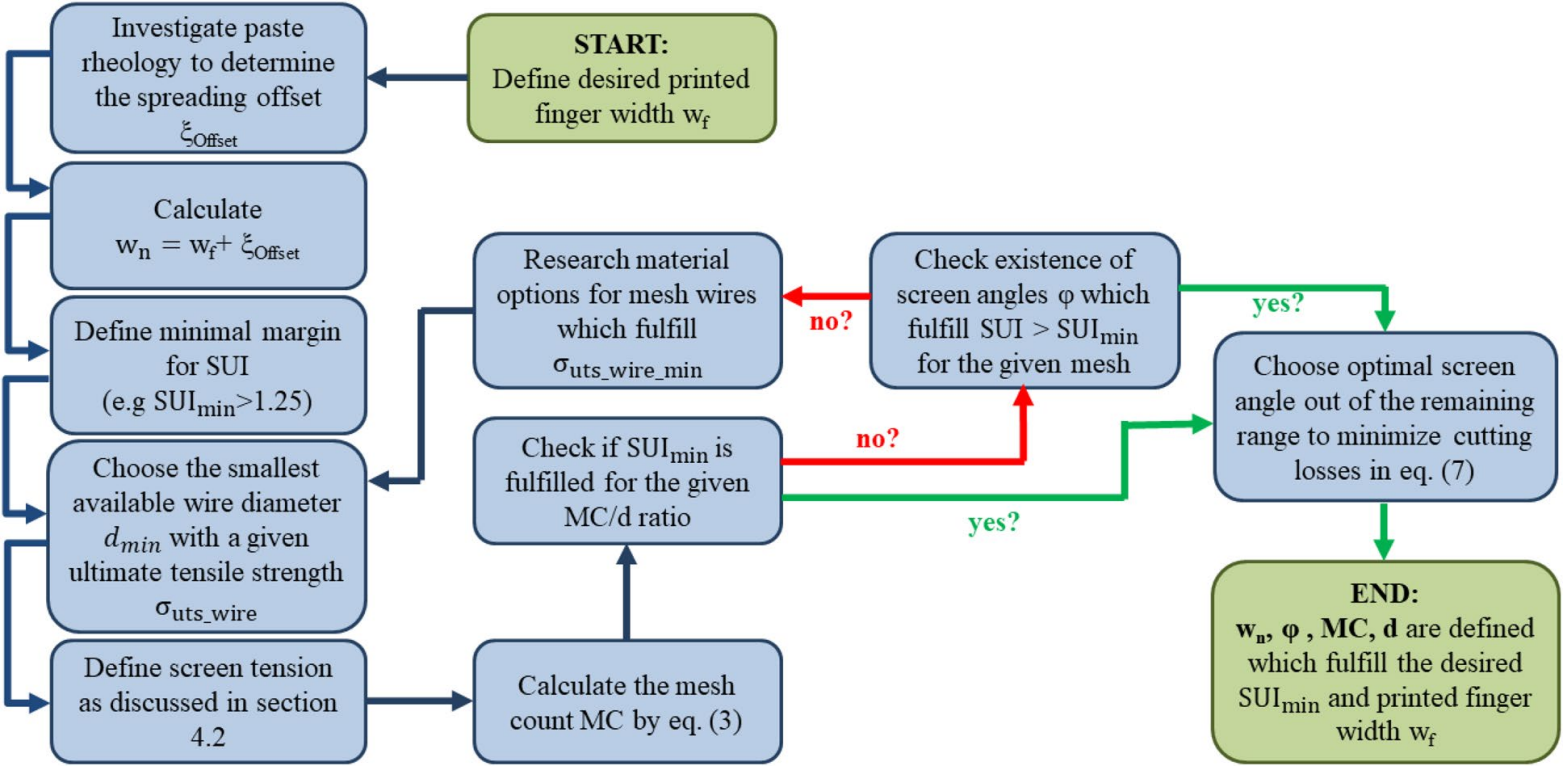
Design approach for the definition of the 2-dimensional screen architecture. In order to reach a desired printed finger width w_f_, a series of design choices has to be made. First, the screen opening width w_n_ needs to be derived by rheological investigation of the paste at the desired printing speed and determination of the expected spreading offset. Furthermore, the margin for the SUI (e.g. SUI_min_ > 1.25), the smallest available wire diameter for mesh production and the desired screen tension γ_screen_ are defined. After calculating the mesh count MC by Eq. (3), the SUI_min_ requirement is controlled. Depending on the result, the screen angle φ is chosen by minimizing the cutting losses, defined by Eq. (9). If no configuration is available which fulfills all requirements, new wire materials need to be developed.

In the previous section, we have discussed how the SUI is dependent on all 2-dimensional screen parameters. Now, we are able to derive a clear approach for designing a screen architecture which will optimize the compromise between expected printability and screen lifetime by using a strong mesh. In Fig. 10, a flow chart is presented which starts at defining the desired goal for a printed finger width w_f_. Afterwards, the spreading offset of a printed finger at the desired printing speed needs to be estimated or analyzed by rheological investigation. Usually, the printed finger width w_f_ is significantly higher than the screen opening width w_n._ For example, if an average printed finger width of w_f_ = 20 µm for industrial printing speeds is desired, one must take spreading in the range of 5 µm into account. After w_n_ is known, the threshold for the minimal SUI_min_ for the desired paste has to be defined. As discussed in section “Correlation of the SUI with screen printing experiments”, the data supports a SUI_min_ = 1.25 for commercial high temperature Ag-paste. Due to practical reasons, the next step should be the choice of the smallest available wire diameter. This decision might be influenced by technical and/or economic reasons. Now, the optimal screen tension is defined by estimation or experience. For mass production of Si-Solar cells a minimum of 20 N/cm should be used^3,14,42^. This value is further used to obtain the optimal mesh count MC by Eq. (3), defining a fixed ratio between MC and d. If this ratio does not fulfill the discussed SUI_min_ requirement, the designer can check if a screen angle is available which pushes the SUI over SUI_min_ requirement. This decision might be further affected by economic reasons due to the angle depending cutting losses of mesh as discussed in section “Simulation of the SUI” and quantified by Eq. (9). If no screen angle exists which fulfills SUI > SUI_min_, the chosen wire material does not offer a solution for the desired printed finger width w_f_ and screen tension γ_screen_. In such a case, the designer has to research for new wire materials or reevaluate its initial technological and economic decision for the smallest available wire diameter.

## Conclusion

Fine line screen printing for solar cell metallization is facing the increasingly difficult challenge of further decreasing the printed finger width to increase cell efficiency and reduce silver consumption per cell. In this study, we present a step by step approach for designing future screen architectures by introducing the novel dimensionless parameter screen utility index SUI. This parameter gives a quantitative indication for the expected printability of any 2-dimensional screen architecture. Further, a full theoretical derivation of the SUI, a correlation to experimental results and an in-depth simulation over a broad range of screen manufacturing parameters is given, revealing a nonlinear relationship to all 2-dimensional screen parameters. This analysis is extended by modeling the angle dependent mesh cutting losses in mass production of screens, giving rise to a classical optimization problem. Finally, we present a prediction for the future of mesh production by simulating the printability of screens made out of novel wire materials (e.g. the use of carbon nanotubes) with very high ultimate tensile strengths in order to fulfill the SUI requirements for printing contact fingers with widths below 10 µm.
